# People-Centred Development of a Smart Waste Bin

**DOI:** 10.3390/s22031288

**Published:** 2022-02-08

**Authors:** Jože Guna, Katarina Polajnar Horvat, Dan Podjed

**Affiliations:** 1 Faculty of Electrical Engineering, University of Ljubljana, 1000 Ljubljana, Slovenia; 2 Research Centre of the Slovenian Academy of Sciences and Arts, Anton Melik Geographical Institute, 1000 Ljubljana, Slovenia; katarina.polajnar@zrc-sazu.si; 3 Research Centre of the Slovenian Academy of Sciences and Arts, Institute of Slovenian Ethnology, 1000 Ljubljana, Slovenia; dan.podjed@zrc-sazu.si

**Keywords:** smart waste bin, user study, IoT

## Abstract

The study presented in this article focuses on the role of a smart waste bin (waste container) designed for waste management and explores what types of interventions people consider more appropriate in promoting environmentally responsible behaviour—based on norms or on an individual’s emotions. The smart waste bin development process was people-centred and paid particular attention to human experiences, allowing for various interaction modalities. By incorporating various sensors for waste volume and weight measurement in conjunction with presence and user identification capabilities, the experience was personalised. User feedback was collected by an extensive survey, consisting of four systematic sections, where values, attitudes, norms, perceived behavioural control, behavioural intention and actual behaviour were examined. The survey was completed by 194 respondents. The results showed that participants at the declarative level show a high level of environmental awareness and are very much willing to handle waste appropriately. Additionally, the results of the R&D process indicated that relatively cheap and efficient technological solutions can be developed to support waste management and sustainable lifestyles if the human-centred approach is taken into account.

## 1. Introduction

The world is increasingly flooded with waste from people’s daily activities which has a significant impact on the environment [1]. Globally, the amount of waste is expanding at an alarming rate. The quantity is closely connected to expanding population, consumption and production patterns [2]. The World Bank predicts that the amount of waste generated annually will increase from 2.01 billion tons in 2016 to 3.40 billion tons in 2050 [3]. In one lifetime, an individual produces on average 600 times as much waste as that person weighs. Daily activities aimed at reducing waste collection in landfills and at recycling and waste prevention can help solve the problem [4]. In doing so, methods of social influence and various types of interventions have proven to be successful in implementing environmentally friendly actions [5,6]. How to address the waste management challenge by a user-centred technology driven solution is the motivation for this research.

The study presented in this article focuses on the role of a smart waste bin (waste container) designed for waste management and explores what types of interventions people consider more appropriate in promoting environmentally responsible behaviour. Although technology driven, the project has been designed with the end user in mind using a user-centred design approach. The user experience, interface and interaction design were especially important topics.

The authors were primarily interested in whether people find a more appropriate intervention based on norms or on an individual’s emotions. Various pro-environmental interventions have been identified, using different techniques, with each focusing on a different set of behavioural determinants [6,7,8,9]. Studies to date have shown that the social and personal norms are the ones that lead more to environmentally friendly behaviour than either an individual’s views, his or her concern about environmental degradation, or his or her environmental awareness [4,10]. Additionally, studies in the field of intervention analysis have shown that those interventions that include a normative component are more efficient than those that involve environmental concerns or financial incentives [10,11]. Interventions based on social and personal norms are efficient since people are most often motivated to act pro-socially [12]; however, what is the impact of interventions based on the emotional component? Previous research in the field of studying environmental behaviour has shown that positive emotions affect a higher willingness to make environmentally friendly donations [13]. Thus, in the present study, the authors were interested in whether positive emotions can also be effective in promoting environmentally friendly behaviour connected to appropriate waste management. Interventions were compared to identify which ones people perceive as more appropriate and would motivate them more in enacting environmentally friendly behaviour, i.e., those based on the normative component or those based on an emotional component. The result was the answer for how the proposed solution aims to tackle the challenges of waste management.

The findings served to develop a smart waste bin, equipped with various sensors. Its main feature is an interaction screen with two different “faces”—one is based on a normative and another on an emotional component. The study was used to design the waste bin and to involve people from different population groups in the design and development of a new technological product. The developed prototype is a direct result of an applied national Slovenian research agency project, titled “The Invisible Life of Waste” [14]. The project involves research academic partners from technical and humanistic backgrounds, as well as a partner from the industry. It is interdisciplinary in nature, providing research and development cooperation opportunities for all partners involved.

## 2. Related Works

### 2.1. People-Centred Development

To address the problem of waste management, the authors of this article decided to rely on people-centred development, which has been tested in several industry settings, from Xerox, Boeing, Microsoft and Nissan, to different EU Horizon 2020 projects, including MobiStyle, TripleA-Reno, INFINITE, re-MARKABLE, U-CERT, NRG2PEERS and the projects, “DriveGreen” and “Invisible Life of Waste”. The key idea of the people-centred development is that people should be involved in the design process.

The approach is usually divided into four steps. The first step is identification, where the R&D team defines the problems that are being solved and who are the people or communities in focus. In the second step, they carry out research and analyse their behaviour, practices, needs and expectations, using and combining different qualitative and quantitative approaches, e.g., interviews, focus groups, participant observation and surveys. The third step is interpretation. On the basis of research findings and in cooperation with the developers and people and the communities in focus, the R&D team prepares recommendations for improving design. The fourth step, testing, assures an optimal user experience. In this step phase, when a prototype of the product or service is developed, the central question is to confirm why and how—or if at all—the solution has been accepted, is relevant, important and meaningful to people [15,16].

One of the core principles of the people-centred approach is to remain in touch with people throughout the R&D process. In the case of this study, the ideal trajectory of the four steps mentioned above was difficult to achieve due to the COVID-19 pandemic. Therefore, the authors of this article had to rely on “contactless” methods and remote research [17], including surveys and videos to introduce the smart waste bin to people in the focus of the study.

### 2.2. User Behaviour

Small individual behaviour changes can lead to a great impact when a significant number of people/users are considered. In [18] the authors use agent-based modelling (ABM) to examine the effectiveness of a nudge policy for improving recycling behaviour. The results based on a real data simulation suggest that nudges, in the form of norm-based policies, can be an effective solution to enhancing people’s recycling behaviour under specific circumstances.

The study in [19] establishes the concept of an IoT-enabled accountability in household waste source separation in China by utilising the lens of the accountability theory. Based on an extensive literature review the authors conclude that a proper application of technology can lead towards the development and adoption of a cleaner production, circular economy and effective waste management, thus improving environmental sustainability.

In [20] the technology acceptance model (TAM), theory of planned behaviour (TPB) and norm activation model (NAM) are used together to analyse college students waste sorting behaviour. The results indicate that perceived ease of use could directly promote waste sorting intentions and behaviour.

In [21] the authors explore the potential of integrating lessons from behavioural sciences to facilitate a circular economy in e-waste management. A review of prominent behavioural theories and their application in the context of sustainable consumption and pro-environmental behaviours is presented.

A survey of waste management in over 220 smart cities is presented in [22]. The results indicate that sustainable cities may seek ways to use the capabilities of disruptive technologies toward making changes in human behaviour to pro-environmental behaviour. 

### 2.3. Smart Waste Management Solutions

For effective people-centred design of solutions, the appropriate selection and application of technologies is essential. The application of ICT, IoT and multimedia technologies are especially important. Thus, the technology acts as an important enabler for providing practical smart waste management solutions and acting towards more sustainable practices.

In [23,24] a literature review for smart waste management and a comparison of the different methodologies is given. The authors focus on the IoT, considering its elements (identification, sensing, communication, computation, semantics, and services) and how the IoT can be used effectively to manage solid waste.

A system for household plastic management through smart bins, A Case of Study in the Philippines, is shown in [25]. It consists of simple smart bins using a weight scale and a smart application that forecasts the amount of waste generated for each bin at different times. Accordingly, it allows for the optimal generation of waste collection routes. The results indicate a positive increase in household plastic waste collection. The presented waste bin solution does not provide any user interaction capabilities, however. 

The research in [26] proposes a hardware and software solution for waste management, including citizens in the process. It is based on the IoT approach, using sensors for waste filling level detection. The results show that the proposed system can change the way people deal with their waste and optimise economic and material resources. Again, no direct waste bin user interaction is possible.

Finally, peoples’ attitudes towards technology use and adoption are essential when trying to change social behaviour. User experience and interfaces play an essential role in the interaction between devices (technology aspect) and people (human aspect). In [27] the authors discuss the importance of human experience and user interface design and interactions for effective waste management in a smart city. The paper focuses on the technology of waste management and the design process to assure an optimal user experience.

## 3. Smart Waste Bin Development

The smart waste bin development process put a particular attention on human experiences, allowing for various interaction modalities. The smart waste bin consists of the following modules: (1) controller, (2) waste measurement sensors, (3) user input interaction and (4) user output interaction modules. 

**Figure 1:**
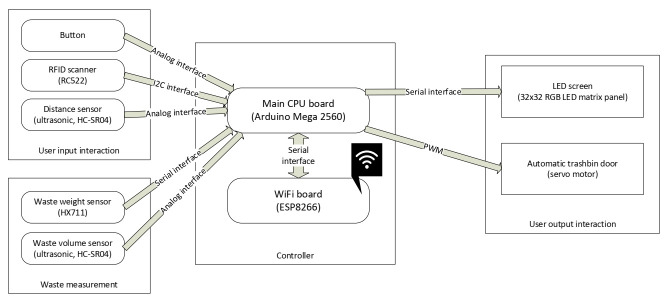
Smart waste bin architecture.

**Figure 2:**
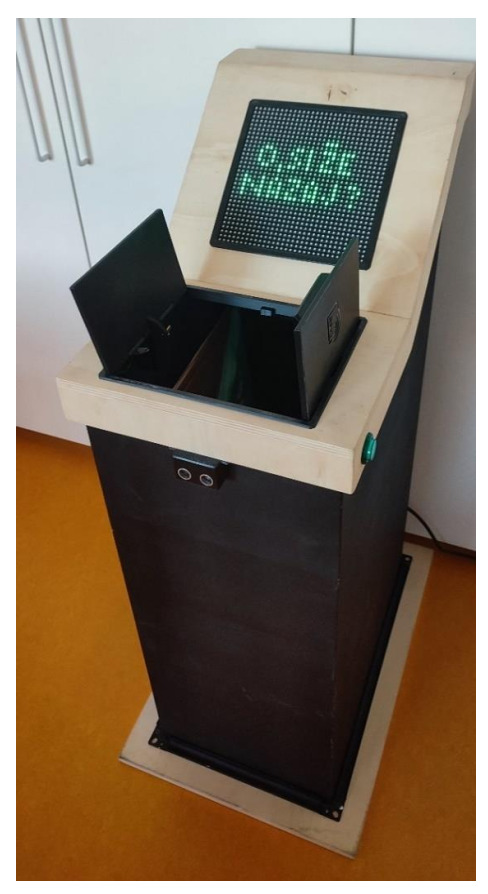
Smart waste bin finished prototype.

The core of the smart waste bin represents the controller, which is based on the Arduino Mega 2560 board in conjunction with the WiFi ESP8266 board. It enables all smart waste bin functionalities by a custom developed software (using Arduino IDE v1.8) and enables backend server wireless communication (IoT aspect). A waste measurement module allows for waste weight (up to 20 kg) and volume measurements (ultrasonic sensor). This information is sent to the cloud and presented to people interacting with the device. User input and output interaction modules provide the interaction capabilities. Different users can be identified by RFID technology (RFID tags or a mobile phone) thus allowing personalization. The user presence is detected by an ultrasonic distance sensor. In this way the smart waste bin is activated automatically, and only when required, thus conserving energy. Additional interaction is possible by two side-placed buttons. Relevant personalised (e.g., waste weight) information is shown on a large 32 × 32 RGB LED matrix display with a “retro” appearance. In this way, a simple graphical feedback is possible, providing the smart waste bin a personal touch by the visualization of a face. The waste bin lid opens automatically and when needed, making it easier to use. The casing of the smart waste bin was custom designed and made from laser-cut metal and wood material, and then enhanced by 3D printed components. The detailed smart waste bin architecture is shown in Figure 1 and the finished prototype in Figure 2.

## 4. Materials and Methods

A smart waste bin prototype was used to answer the main research question, i.e., whether a normative or an emotional approach is better to motivate people toward a more sustainable waste management behaviour. 

The smart waste bin automatically detects the user presence (ultrasonic proximity sensor) and allows for the personalisation of interactions by the RFID identification. Disposed waste is weighed and measured in volume, and the results presented on a “retro” matrix LED screen. The smart waste bin has the visualisation of eyes on the screen and other human features, since people like to anthropomorphise animals, plants and things and may therefore be more receptive to messages about waste as a significant negative factor in the global network of production and consumption. 

### 4.1. Evaluation Scenarios

**Figure 3:**
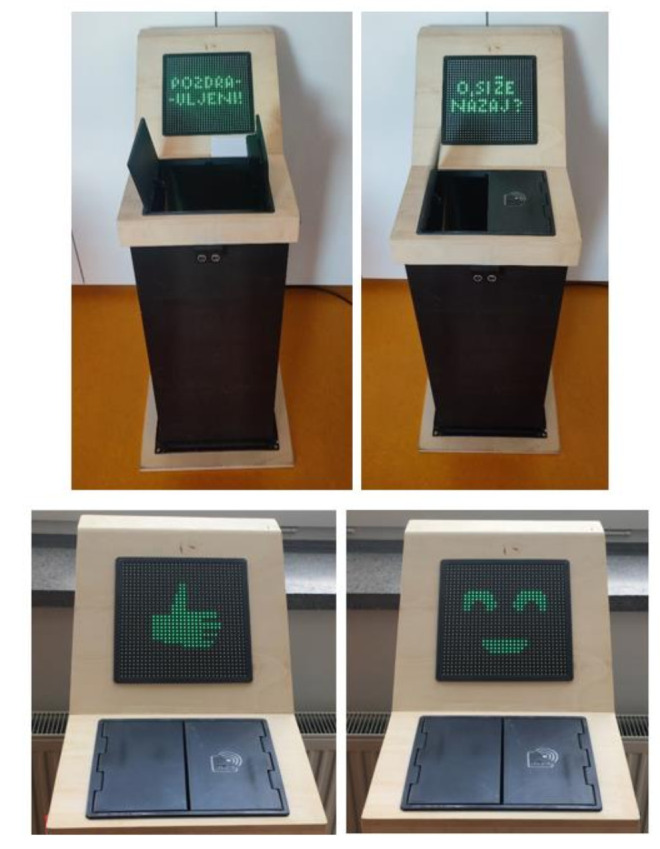
Two visualisations (“faces”) of the smart waste bin—normative (**left**) and emotional (**right**).

We created two scenarios, one focusing on a normative representation of waste management information (Figure 3 left) and the other on emotional information (Figure 3 right). The waste bin in the normative information scenario first winks and greets an individual with a formal greeting, “Hello”, when approached. After the person has disposed of the waste and obtained information about its weight, the waste bin provides the statement “In LJ 61% of people separate their waste” (“in LJ” meaning “in Ljubljana”) and it then shows the “thumbs up” sign. The waste bin in the “emotional” scenario greets the individual with a personal, informal greeting, “Oh, you are back?”. When the person has disposed of the waste and obtained information about its weight, the waste bin provides the following statement: “I am glad you separate your waste”. Finally, a smiling face is shown on the screen.

### 4.2. Evaluation Method and Procedure

Testing of both versions of the interface was conducted online due to the national and international measures related to the COVID-19 pandemic. In parallel to the testing of both scenarios, an extensive online survey was carried out, where one of the key questions asked which of the two respondents would be considered to be more appropriate in connection to waste management. The survey was carried out in spring 2021.

Both scenarios were presented as videos and shared on the YouTube platform to enable easier access. Before completing the survey, each individual was invited to observe carefully the two scenarios on the normative and emotional aspect and then to complete the survey. We used an open-source platform, www.1ka.si (accessed on 1 December 2021) [28], to conduct the survey. The online survey was conducted over a four-month period and was aimed at the population of the whole country, i.e., Slovenia. The study was counterbalanced in terms of scenario order selection.

The survey consisted of four systematic sections, where values, attitudes, norms, perceived behavioural control, behavioural intention and actual behaviour were examined. The participants in the research evaluated the selected statements which measured selected factors on the basis of the Likert scale (1—completely insignificant, 2—insignificant, 3—medium, 4—important, 5—very important). The higher they ranked them on the scale, the more important they seemed in their lives. 

The values were measured with ten quality-of-life indicators determined by the Schwartz scale of values [29] and by the results of selected research on the impact of values on environmental behaviour [30,31]. The quality-of-life indicators were as follows: (1) equality and justice: equal opportunities for all, redressing injustices; (2) peace: a state free of wars and tensions united in altruistic values; (3) power and influence: striving to assert one’s will, controlling others; (4) ambition: diligence; (5) wealth: material possessions, money, living in abundance; (6) reputation and fame: being recognised, established in a society; (7) united in egoistic values; comfort and enjoyment: to live relaxed, personal comfort, satisfaction of desires; joy of life: to enjoy food, leisure …; (8. which are united in hedonic values; (9) coexistence with nature: to adapt to nature, to live in accordance with the principles of sustainable development; and (10) protecting the environment: to take care of the preservation of the natural environment, to prevent pollution; which are united in biospheric values. In studying values, we proceeded from the assumption that values form several common dimensions [29], which we then combined into individual constructs. 

The reliability of the individual construct was checked with the Cronbach’s alpha coefficient. Cronbach’s alpha was 0.62 for altruistic values, 0.64 for egoistic values, 0.80 for hedonic values, and 0.63 for biospheric values. We measured attitudes with the help of four statements: “I am a person who handles waste properly”, “Improper waste management is a big social problem”, “Waste needs to be treated properly”, and “Appropriate management of waste today is crucial”. Their Cronbach’s alpha was 0.75. We measured personal norms with the following statements: “Proper waste management is an important part of me”, “I feel guilty if I do not handle waste properly”, “I am proud if I handle waste properly”, and “I feel moral responsibility for the proper management of waste”. The Cronbach’s alpha was 0.85. We measured subjective norms with two statements: “My relatives believe that waste should be treated properly” and “I take into account the views of my loved ones on waste management”. The Cronbach’s alpha was 0.76. Perceived behavioural control was measured by the statements “I know a lot about proper waste management” and “Proper waste management seems too difficult to me”, with a Cronbach’s alpha of 0.52. We measured the intention for environmentally friendly behaviour with the statement “I am willing to handle waste even more conscientiously”, and the behaviour with the statement “I regularly separate waste in my household and I strive to produce as little of it as possible”. The main section was designed to study the evaluation of the two aspects presented. We measured the preferred waste management handling with the question, “What convinces you to handle waste properly?” and the possible answers of: “concrete data” or “emotional address”. We also measured demographic factors with standard scales for gender, age, education, and current employment status.

## 5. Results and Discussion

### 5.1. Study Participants

The survey was completed by 194 respondents. Of these, 71% were women, 27% were men and 2% did not wish to identify themselves by gender. In terms of age structure, the majority of respondents were aged between 41 and 60 years old with 39%, followed by those aged between 21 and 40 years old with 33%, those aged 61 and over with 19% and those aged under 20 years old with 8%. In terms of educational structure, the majority of respondents were highly educated, with 84% having a Master’s or PhD degree, a post-secondary education, a college or a university degree, while only 8% having a secondary education, 1% having a vocational education and 7% having a primary education. The survey was mainly responded to by the active population, i.e., employed or working people.

### 5.2. Survey Results

In the research, we were interested in the extent to which participants were willing to act in an environmentally friendly manner and to what extent they actually do so. The results showed that participants at the declarative level showed a high level of environmental awareness and were very much willing to handle waste appropriately, where the mean value was 4.56. This was not surprising, since people often express a high level of awareness (or, as Beckmann [32] wrote, “who would actually dare to admit a lack of interest in environmental problems or an environmentally unfriendly attitude?”). One of the proofs of this arose quickly—the impression of a positive attitude towards the environment began to fade when participants were asked about actual environmental action. The average value of the demonstrated actual environmental position was 4.31. The transition to one’s own active participation in solving environmental problems thus expressed the gap between those who expressed their commitment to environmental protection only at the level of attitudes and those for whom caring for the environment actually held value (Table 1). This result is largely consistent with the results of similar research to date on the gap between environmental awareness and behaviour [4,33,34]. The Table 1 shows the willingness to behave in an environmentally friendly manner on a hypothetical and active level where the respondents (N = number of respondents) answered how much they agreed with the statement with the help of a Likert scale (1—completely insignificant, 2—insignificant, 3—medium, 4—important, 5—very important).

**Figure 4:**
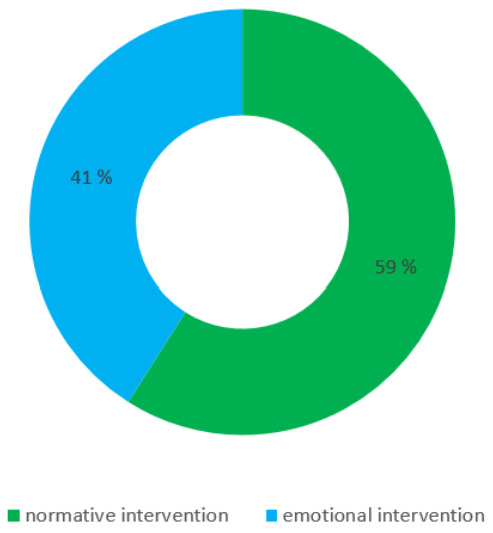
Normative vs. emotional information presentation.

The results of the survey (Figure 4) showed that people rate the waste bin with normative information as more suitable for waste management with 59%, and the waste bin with emotional information as less suitable by 41% of correspondents.

Based on the t-test of independent samples, we found that responses of the study participants regarding the choice of normative (index 1) and emotional (index 2) intervention in terms of their gender, age and educational structure did not differ statistically significantly, however, certain interesting differences between the individual groups of participants were indicated. Regarding the gender structure, both genders evaluated the choice of the type of intervention practically equally. Both genders largely preferred a normative intervention with a mean value of 1.40 (mean of the indexes 1 and 2) for women and 1.41 for men (Table 2).

However, younger respondents rated higher the waste bin with the emotional (index 2) and more personal appeal, while older respondents rated higher the one with the normative appeal (index 1). Under 20-yearsold respondents rated the waste bin with the emotional intervention as more appropriate for promoting waste management, on an average 1.56 (mean of the indexes 1 and 2). Those aged 21 to 40 rated the normative intervention as more appropriate on an average 1.44. Participants aged 41 to 60 (on an average 1.37), and over 61 (on an average 1.38) rated the normative version of the waste bin higher than the emotional version (Table 3).

In terms of education, those with a university or higher education rated the normative waste bin higher than the older ones, with on average 1.38 (mean of the indexes 1 and 2). Those with a secondary education rated the emotional appeal more highly with on average 1.60. Those with a vocational education were completely unanimous in their preference for the emotional appeal waste bin, while those with a primary education also rated the emotional appeal higher than the normative one, with an average of 1.57 (Table 4).

In studying the role of individual constructs of psychological variables, values, attitudes, personal norms, subjective norms and perceived behavioural control in the choice of normative or emotional intervention, we found that within the values, altruistic and biosphere values were those that influenced the choice of normative intervention. Conversely, hedonic and biospherical values were the ones that most influenced the choice of emotional intervention. This result is not surprising, as hedonic values are those that are reflected in positive and emotionally related orientations, such as a joy of life, comfort and enjoyment [35]. Biosphere values, on the other hand, are linked to orientations such as a coexistence with nature, environmental protection, and are thus surprisingly positively linked to appropriate waste management, regardless of the intervention we used, whether emotionally or normatively. On the other hand, altruistic values, which are reflected in orientations such as equality and justice, are more closely linked to social and personal norms, collective social orientations and concern for the well-being of all people in the world. According to attitudes, there were no significant differences in their impact on selection. On the other hand, personal and subjective norms had a more visible influence on the selection. Thus, those who preferred normative intervention valued personal norms and subjective norms to a greater extent. Which is not surprising, since human norms are reflected in an individual’s sense of moral duty to act in an environmentally friendly manner and relate to their perception of what is “appropriate” in a given situation [36]. The participants also highly valued perceived behavioural control, which was reflected in their own ability to act in an environmentally friendly manner. The reason for this may be that normative intervention, when communicating certain information to participants, in our case the amount of separately collected waste in Ljubljana, encourages people to feel their own ability and a sense of higher capacity for environmentally friendly behaviour (Table 5). In terms of intention and actual behaviour, the results show that those with a higher intention to act environmentally friendly and actually behave in this way were more likely to choose the normative intervention.

### 5.3. Qualitative Findings

From the research point of view, the section of the questionnaire where the participants could freely express their opinions was similarly important as it served as a basis for further ethnographic work and in-depth discussions. In this way, the researchers obtained much more qualitative data than in a workshop in which only a limited number of participants would have taken part. In addition to the quantitative data, the research team received 31 pieces of written feedback that were analysed and categorised into three categories: positive, neutral, and negative. The majority of the feedbacks (18 responses) were neutral, with 11 negative, and 2 positive. The main objection of the negative feedback was that the research and development team had, as expressed by one person, “designed yet another electronic and plastic device that will eventually become a problematic piece of waste.” Other respondents also stated in their negative feedback that they disliked the “use of electronics for motivational words” and saw it as a “waste of energy, materials and time.” It could be used, as the same person explained, “to motivate children and not adults.”

The more constructive negative feedback included specific suggestions for improving the device. For example, respondents said that the waste management process, enabled by the smart waste bin, was relatively slow. In their opinion, the lid opening system should be faster to make the recycling process more efficient and people-friendly. One of them also mentioned that people might be afraid of the device that measures the weight, as the price of their waste might be calculated according to its weight. At least three people (one from the negative and two from the neutral response spectrum) also suggested combining the different addresses of the bin, as people need both at the same time, i.e., “the objective data (reason) and the subjective content (emotions).” One of them even presented a possible scenario that would merge the two options:

“In the first phase (when the card is placed on the sensor): ‘Hello, I collect organic waste, e.g., packaging and paper.’ When the flaps open: ‘Do you know that/insert an interesting fact/’? And finally, just before the flaps close: ‘Thank you for CORRECT waste management’ or ‘Thank you for CORRECT SORTING of waste’ or something similar.”

Such concrete suggestions were particularly useful for improving the solution and finding new ways for people and technology to interact with people and their communities.

### 5.4. Comparison to Other Studies

Smart technology and human-centred waste management is important for modern cities, as shown in [22]. A combination of technology and especially human oriented solutions, specifically focusing on user experience and interaction design, can potentially change individual habits that can lead to large pro-environmental changes [18,19,20,21]. 

Our study specifically addresses the challenge of whether positive emotions [13] can also be effective in promoting environmentally friendly behaviour connected to appropriate waste management. By designing two different user interfaces, we investigated which kind of interaction would motivate the users more towards this goal—those based on the normative component or those based on an emotional component. Studies to date have shown that the social and personal norms are the ones that lead more to environmentally friendly behaviour than either an individual’s views, his or her concern about environmental degradation, or his or her environmental awareness [4,10].

Considering the technical implementation, existing studies are somewhat lacking in the user experience and interaction aspects. In [18,19,20,21], comparable smart waste management technologies and solutions are given; however, the user aspects are lacking.

### 5.5. Study Limitations

The testing of the device was originally supposed to take place in workshops, at which people from various social and age groups would gather and participate in the design of a new solution. Since such workshops could not be physically organised due to the COVID-19 lockdown and other restrictions in the second half of 2020, researchers turned instead to digital solutions. Scenes of the interaction of a researcher with two variants of the waste bin were recorded, the two videos were uploaded on YouTube and disseminated online with a request for people to view them and fill in the attached questionnaire, in which there was also a section in which the respondents were able to explain more extensively and freely what they thought about the device and its interface (for limitations during COVID-19 crisis and lockdown in this and other projects see [37]). Since the study was carried out online instead of physically (in a real environment), the participants were limited to those using: (1) online social networks, and (2) YouTube or similar digital tools. In addition, the study potentially excluded certain user groups, e.g., elderly people. Nevertheless, the study included a large number of participants (194), thus still providing relevant results.

## 6. Conclusions

The results of the R&D process show that relatively cheap and efficient technological solutions can be developed to support waste management and sustainable lifestyles if the human-centred approach is taken into account. In the case presented in this article, the approach was used to tailor the IT-based solution, i.e., a smart waste bin, to different needs and requirements of people. Two different versions of the user interface were designed and tested with people. The results show that the universal (“one-size-fits-all”) approach is not suitable for people with different values, mind-sets and habits.

The emotional intervention was found to be more suitable for young and moderately educated people and the ones with higher hedonic and at the same time biospheric value orientations. On the other hand, a normative-based intervention was found to be more suitable for mature, older and highly educated people. They value more altruistic and biospheric views and at the same time highly value personal and subjective norms. They also highly value perceived behavioural control, meaning that they are more aware of their own abilities or feel qualified to implement environmentally friendly behaviour towards waste management. Overall, normative interventions have proven to be more successful in promoting environmentally friendly behaviour, but an in-depth interdisciplinary approach is still needed in the selection of more specific interventions.

On the basis of the findings presented in this article, we propose to use a tailored approach focused on a combination of engineering and social sciences in the further development of similar solutions to motivate people to change their habits towards a more sustainable behaviour. Moreover, the research and development teams could consider a wider variety of people and design multiple versions of the user interface to achieve a better impact.

A similar approach could be used to design several other similar user interfaces. For example, it could be used to design interfaces in vehicles, which would be tailored to the driver and his or her personality. It could be also used in smart homes for home devices, such as thermostats, ovens, refrigerators, washing machines, laundry machines and other devices using energy. When a specific system provides value to the user and is simple and enjoyable to use, small changes (e.g., energy consumption optimisation) on the individual scale can provide a large impact on the large scale. Thus, the importance of a proper combination of technology and a tailored user experience is revealed.

The approach could also be particularly relevant for designing pedagogical solutions to provide more personalised pedagogical methods and to educate students about a sustainable lifestyle. 

## Figures and Tables

**Table 1:** Willingness to behave in an environmentally friendly manner on a hypothetical and active level.

Statement	N	1	2	3	4	5	Mean Value	Standard Deviation
“I am willing to handle waste even more conscientiously”	193	0.5%	0%	8.3%	24.9%	66.3%	4.56	0.69
“I regularly separate waste in my household and I strive to produce as little as possible.”	194	0.5%	1.0%	9.3%	44.9%	44.3%	4.31	0.726

**Table 2:** Gender and normative vs. emotional information presentation (index 1 = normative intervention, index 2 = emotional intervention).

Gender	N	Mean Value	Standard Deviation
female	53	1.40	0.494
male	136	1.41	0.494
Total	189	1.41	0.494

**Table 3:** Age and normative (index 1) vs. emotional information (index 2) presentation.

Age	N	Mean Value	Standard Deviation
to 20 years old	16	1.56	0.512
from 21 to 40 years old	64	1.44	0.500
from 41 to 60 years old	76	1.37	0.486
more than 61 years old	37	1.38	0.492
Total	193	1.41	0.493

**Table 4:** Education and normative (index 1) vs. emotional (index 2) information presentation.

Education	Mean Value	N	Standard Deviation
primary school	1.57	14	0.514
vocational education	1.00	1	
secondary education	1.60	15	0.507
university or higher education	1.38	163	0.487
Total	1.41	193	0.493

**Table 5:** Psychological factors, intention, behaviour and normative (index 1) vs. emotional (index 2) information presentation.

Constructs		N	Mean Value	Standard Deviation
altruistic values	normative intervention	114	4.88	0.29
	emotional intervention	79	4.75	0.43
egoistic values	normative intervention	114	2.72	0.72
	emotional intervention	79	2.80	0.68
hedonic values	normative intervention	114	4.14	0.69
	emotional intervention	79	4.35	0.61
biospheric values	normative intervention	114	4.62	0.56
	emotional intervention	79	4.66	0.56
attitudes	normative intervention	114	4.59	0.47
	emotional intervention	79	4.60	0.46
personal norm	normative intervention	114	4.45	0.59
	emotional intervention	79	4.21	0.76
subjective norm	normative intervention	114	4.07	0.86
	emotional intervention	79	3.78	0.72
perceived behavioural control	normative intervention	114	4.21	0.58
	emotional intervention	79	4.12	0.59
actual behaviour	normative intervention	114	4.32	0.72
	emotional intervention	79	4.29	0.74
behavioural intention	normative intervention	114	4.57	0.73
	emotional intervention	79	5.56	0.63

## Data Availability

Data is available upon request.
